# Supplementary material for: Iterative improvement in the automatic modular design of robot swarms
Source: PeerJ Comput Sci. 2020 Dec 7;6:e322. doi: 10.7717/peerj-cs.322 (PMC7924708; doi:10.7717/peerj-cs.322)
Supplement: Supplemental Information 3 [file peerj-cs-06-322-s003.zip › argos3/doc/api/standalone/a00399.html]

ARGoS: core/utility/plugins/factory.h File Reference


- Main Page
- Related Pages
- Namespaces
- Classes
- Files

- File List
- File Members

# core/utility/plugins/factory.h File Reference

`#include <argos3/core/utility/configuration/argos_exception.h>`  
`#include <map>`  
`#include <iostream>`  
`#include <string>`  
`#include <cstdlib>`  
`#include <argos3/core/utility/plugins/factory_impl.h>`  

Include dependency graph for factory.h:

Go to the source code of this file.

|  |  |
| --- | --- |
| Classes | |
| class | argos::CFactory< TYPE > |
|  | Basic factory template. More... |
| struct | argos::CFactory< TYPE >::STypeInfo |
|  | A struct containing the information about the registered types. More... |
| Namespaces | |
| namespace | argos |

|  |  |
| --- | --- |
|  | The namespace containing all the ARGoS related code. |

| Defines | |
| #define | REGISTER\_SYMBOL(BASECLASS,CLASSNAME,LABEL,AUTHOR,VERSION,BRIEF\_DESCRIPTION,LONG\_DESCRIPTION,STATUS) |
|  | Macro to register a symbol into the factory. |

---

## Define Documentation

|  |  |  |  |  |  |
| --- | --- | --- | --- | --- | --- |
| #define REGISTER\_SYMBOL | ( | BASECLASS, |  |  |  |
|  |  | CLASSNAME, |  |  |  |
|  |  | LABEL, |  |  |  |
|  |  | AUTHOR, |  |  |  |
|  |  | VERSION, |  |  |  |
|  |  | BRIEF\_DESCRIPTION, |  |  |  |
|  |  | LONG\_DESCRIPTION, |  |  |  |
|  |  | STATUS |  | ) |  |

**Value:**

```
namespace argos {                                                    \
      extern "C" {                                                      \
         BASECLASS* BASECLASS ## CLASSNAME ## Creator() {               \
            return new CLASSNAME;                                       \
         }                                                              \
      }                                                                 \
      class C ## BASECLASS ## CLASSNAME ## Proxy {                      \
      public:                                                           \
      C ## BASECLASS ## CLASSNAME ## Proxy() {                          \
         CFactory<BASECLASS>::                                          \
            Register(LABEL,                                             \
                     AUTHOR,                                            \
                     VERSION,                                           \
                     BRIEF_DESCRIPTION,                                 \
                     LONG_DESCRIPTION,                                  \
                     STATUS,                                            \
                     BASECLASS ## CLASSNAME ## Creator);                \
      }                                                                 \
      };                                                                \
      C ## BASECLASS ## CLASSNAME ## Proxy BASECLASS ## CLASSNAME ## _p; \
   }
```

Macro to register a symbol into the factory.

**Parameters:**
:   |  |  |  |
    | --- | --- | --- |
    |  | *BASECLASS* | The base class of the hiearchy of types to register (corresponds to `TYPE` in the factory) |
    |  | *CLASSNAME* | The actual class to register |
    |  | *LABEL* | The label to associate to `CLASSNAME` |
    |  | *AUTHOR* | The author of the class `CLASSNAME` |
    |  | *VERSION* | Version information for the code in `CLASSNAME` |
    |  | *BRIEF\_DESCRIPTION* | A brief description of what `CLASSNAME` is |
    |  | *LONG\_DESCRIPTION* | A detailed description of what `CLASSNAME` is and does |
    |  | *STATUS* | Status information (usable, draft, todos, etc) for the code in `CLASSNAME` |

Definition at line 156 of file factory.h.

---

Generated on 10 Jul 2018 for ARGoS by 
 1.6.1
